# Empowering parents for human immunodeficiency virus prevention: Health and sex education at home

**DOI:** 10.4102/sajhivmed.v21i1.970

**Published:** 2020-06-29

**Authors:** Taygen Edwards, Ntombizodumo Mkwanazi, Joanie Mitchell, Ruth M. Bland, Tamsen J. Rochat

**Affiliations:** 1 Africa Health Research Institute, Somkhele, South Africa; 2 Liggins Institute, University of Auckland, Auckland, New Zealand; 3 Human and Social Capabilities Division, Human Sciences Research Council, Durban, South Africa; 4 DSI-NRF Centre of Excellence in Human Development, Faculty of Health Sciences, University of the Witwatersrand, Johannesburg, South Africa; 5 Lentegeur Psychiatric Hospital, Department of Health, Government of the Western Cape, Cape Town, South Africa; 6 Royal Hospital for Sick Children, Glasgow, Scotland; 7 Institute of Health and Wellbeing, University of Glasgow, Glasgow, Scotland; 8 School of Public Health, University of the Witwatersrand, Johannesburg, South Africa; 9 SAMRC Developmental Pathways to Health Research Unit (DPHRU), Faculty of Health Sciences, University of the Witwatersrand, Johannesburg, South Africa

**Keywords:** health education, sex education, intervention materials, HIV prevention, HIV-uninfected children, parent–child communication

## Abstract

**Background:**

Improving health literacy amongst human immunodeficiency virus (HIV)-positive mothers could strengthen child and adolescent HIV prevention. The Amagugu intervention included health literacy materials to strengthen maternal communication and has demonstrated success in low-resource HIV-endemic settings.

**Objectives:**

Our aims were to (1) evaluate whether Amagugu materials improved health literacy leading to changes in parental behaviour towards communicating on topics such as HIV, health behaviours and sex education, and (2) explore what additional information and materials mothers would find helpful.

**Method:**

The Amagugu evaluation included 281 HIV-positive mothers and their HIV-uninfected children (6–10 years). Process evaluation data from exit interviews were analysed using content analysis and logistic regression techniques.

**Results:**

Of 281 mothers, 276 (98.0%) requested more educational storybooks: 99 (35.2%) on moral development/future aspirations, 92 (32.7%) on general health, safety and health promotion, and 67 (23.8%) on HIV and disease management. Compared to baseline, mothers reported that the materials increased discussion on the risks of bullying from friends (150; 53.4%), teacher problems (142; 50.5%), physical abuse (147; 52.3%) and sexual abuse (126; 44.8%). Most mothers used the ‘HIV Body Map’ for health (274; 97.5%) and sex education (267; 95.0%). The use of a low-cost doll was reported to enhance mother–child communication by increasing mother–child play (264; 94.3%) and maternal attentiveness to the child’s feelings (262; 93.6%).

**Conclusion:**

Parent-led health education in the home seems feasible, acceptable and effective and should be capitalised on in HIV prevention strategies. Further testing in controlled studies is recommended.

## Introduction

Prevention of paediatric and adolescent human immunodeficiency virus (HIV) is a global priority.^1^ In South Africa, substantial investment in reducing vertical transmission of HIV has led to more children being born HIV-negative.^2^ However, the rate of new infections, particularly amongst adolescents, remain high.^3,4^

Children who are HIV-negative but have an HIV-positive parent are especially vulnerable. Some evidence has shown that parental HIV may be associated with increased sexual risk behaviour and HIV infection amongst HIV-exposed or HIV-affected adolescents.^5,6^ Although the process by which these risks may be conferred (i.e. parenting, parental illness, parental death) is still unclear, it is plausible, given international evidence, that at least some of these effects may occur as a consequence of parenting capacity or the absence thereof.^7,8,9,10^ The proportion of South African children who live with a parent infected with HIV is large^4^ and the burden on the South African health system is high,^11^ thus limiting the feasibility of providing parental health literacy training in primary health or HIV treatment care settings.^12^ Therefore, it is important to consider task shifting to lay workers as a means to deliver training and education that enable HIV-positive parents to use developmentally appropriate health literacy strategies in the home setting.^11^ In turn, this could increase preadolescent children’s capacity to remain free from HIV in later life.^10^ Utilising caregivers to strengthen HIV prevention in the home during the preadolescent years, before the onset of the high-risk adolescent period, has substantial potential but has been underexploited and under-researched in South Africa.

In South Africa, the predominant HIV health literacy strategies are implemented in school-based education models which tend to target older children aged 14–16 years.^13^ These strategies have been moderately successful in targeting HIV risk behaviours and increasing HIV-related knowledge in low- and middle-income countries.^14^ However, the quality and delivery of HIV, health and sex education in rural South African schools are considered to be poor, and inconsistent, as the teachers themselves often lack the capacity to fulfil this educational role.^15^ Evidence has shown that targeting the caregiver in the home setting is a cost-effective response to increasing health education, in particular, in resource-scarce settings.^16^ Globally, interventions which target parents in the home are on the increase, for example, countries such as France, the Netherlands, Australia and the United States are increasingly focused on parental capacity as a strategy for promoting healthy ideas around reproductive health in young people.^17,18^ Research to date has shown that caregivers have an influential role in HIV prevention and in ensuring the optimal development of children.^8,18^

Central to achieving increased health promotion by caregivers is providing them with the necessary skills and training. Health promotion is broadly defined as a process of enabling people to increase control over and improve their health.^19^ The World Health Organisation includes three key elements in its definition of health promotion: (1) governance to ensure the removal of structural barriers to adequate access to health; (2) healthy cities which limit geographical hindrances to health and (3) most relevant to this study is to ensuring health literacy. Health literacy refers to the knowledge, skills and information individuals need to make healthy choices and central to improving health literacy is ensuring that individuals have the capacity to obtain, process and understand health information.^20^ More recently, the literature has begun to emphasise the need to go beyond simply providing health information and to move towards ensuring capacity to change behaviour, which from a psycho-social perspective is critical.^21^

However, very little is available to support parent’s health literacy, with many parents reporting that they feel ill-equipped to provide education to their children with HIV or sex education.^22^ In response, the South African National Strategic Plan for HIV, tuburculosis (TB) and sexually transmitted infections (STI) 2017–2022 has included a focus on early parenting interventions to support resilience in children.^23^ However, these focus predominantly on the early years, while little is known about the support needs of caregivers in contexts of parental HIV in South Africa, and almost no interventions have focused on primary school-aged children.^11,24^ One maternal HIV-disclosure intervention (Amagugu) focused on supporting maternal disclosure to HIV-uninfected primary school-aged children has been shown to be effective in improving mother-led health behaviours and health promotional activities (such as taking children to clinic to learn about health services); in improving maternal HIV-disclosure rates; and in strengthening the quality of the mother-child relationship in South Africa.^11,24,25,26^ This manuscript undertakes a detailed analysis of the Amagugu process evaluation with the aims of (1) evaluating whether Amagugu materials improved health literacy leading to changes in parental behaviour towards communicating on topics such as HIV, health behaviours and sex education, and (2) identifying what additional informational needs (over and above existing Amagugu content) might be helpful for parents.

## Methods

### Study design and intervention

The Amagugu intervention was based on a health literacy and promotion conceptual framework that was informed by an extensive body of literature.^10^ In summary, the conceptual framework hypothesised that the relationship between parental HIV and child outcomes was mediated through parenting and that parent–child communication is central to improving parent-led health promotion. Specifically, Amagugu hypothesised that non-disclosure and avoidant coping leads to low-quality parent–child communication which, in turn, decreases the likelihood of health and sex education. These children then enter adolescence with a diminished capacity for healthy behaviours resulting in increased risk-taking and adverse outcomes such as HIV infection. The conceptual framework has been described in detail elsewhere.^10^

**FIGURE 1:**
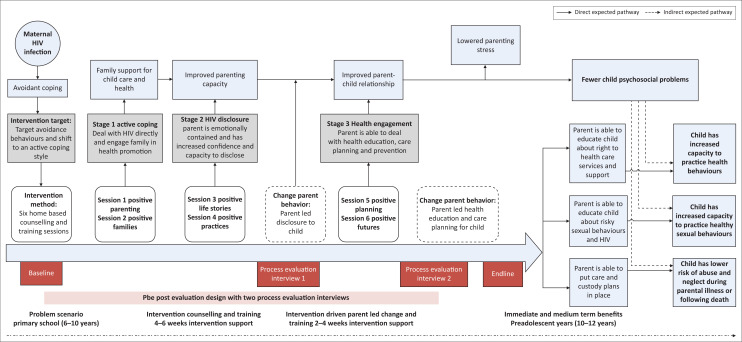
The Amagugu intervention materials sourced from and reproduced with permission from the authors.^10^

The intervention model (see Figure 1) was designed to disrupt these risk pathways by targeting the avoidant behaviour, facilitating maternal HIV-disclosure thereby improving parent–child communication, and fostering parent-led health education, engagement with primary healthcare services and custody planning for their child. Mothers were supported to disclose their HIV status to their child at a level with which they felt comfortable. This included either ‘partial disclosure’ using the word ‘virus’, or ‘full disclosure’ using the word ‘HIV’.^25,27^

Amagugu involved six home-based sessions delivered by experienced lay counsellors.^11,25^ Lay counsellors had completed high school, had previous counselling experience and were trained on Amagugu.^11^ The intervention package (Figure 1) included low-cost, age-appropriate materials that were given to the mother to use with her child. The health literacy materials supported HIV disclosure and education, health information on the importance of nutrition, hygiene, physical activity and health promotion activities which encouraged parental engagement with health services through a series of parent-led activities undertaken in a primary healthcare setting.^10^ A central part of the Amagugu approach involved training parents in health literacy and encouraging change in parental behaviour towards health promotion and communication. As such, counsellors did not interact directly with children, but rather the mothers were trained on the use of the materials so that they could lead the activities with their child without the involvement of the counsellor. This aimed to ensure the transfer of learning, encourage behaviour change and increase parental confidence.

Each mother–child pair received one intervention package consisting of 17 materials. The health literacy materials included a variety of activity cards, educational games and storybooks, such as the ‘Family Treasures Story Book’, an illustrated 14-page English-isiZulu storybook designed to foster closeness between the mother and child; the ‘Disclosure Safety Hand’, which served as a tool to create a confidante circle for the child that helped discourage the child’s disclosure of maternal HIV status to others beyond her confidante circle, in a way easily understood by the child. The tool also encouraged the child to feel safe to disclose any risks at home or school to ‘safety hand’ adults in the household; an ‘HIV Body Map’, a tool for sex and health education including how to explain HIV to a child; and a culturally appropriate doll which facilitated play and parent–child communication. Non-index children in a household were also given a doll. The families were able to keep the intervention materials after the intervention had ended.^11^

Amagugu has been implemented successfully in a pilot study with 24 mothers^11^; in a large-scale evaluation with 281 mothers^25,26^ and in a randomised controlled trial with 464 mothers.^24^ This analysis used data from the large-scale evaluation; specifically, the process evaluation data collected during the exit interviews with the 281 mothers.

### Study setting and population

This study was conducted between 2010 and 2012 at the Africa Health Research Institute, previously known as the Africa Centre for Population Health (‘Africa Centre’), situated in a rural community in northern KwaZulu-Natal with a high HIV prevalence rate.^28^ A Prevention of Mother-to-Child Transmission (PMTCT) programme was implemented in 2001,^29,30^ followed by a decentralised HIV treatment and prevention programme in 2004.^30,31,32^

**FIGURE 2:**
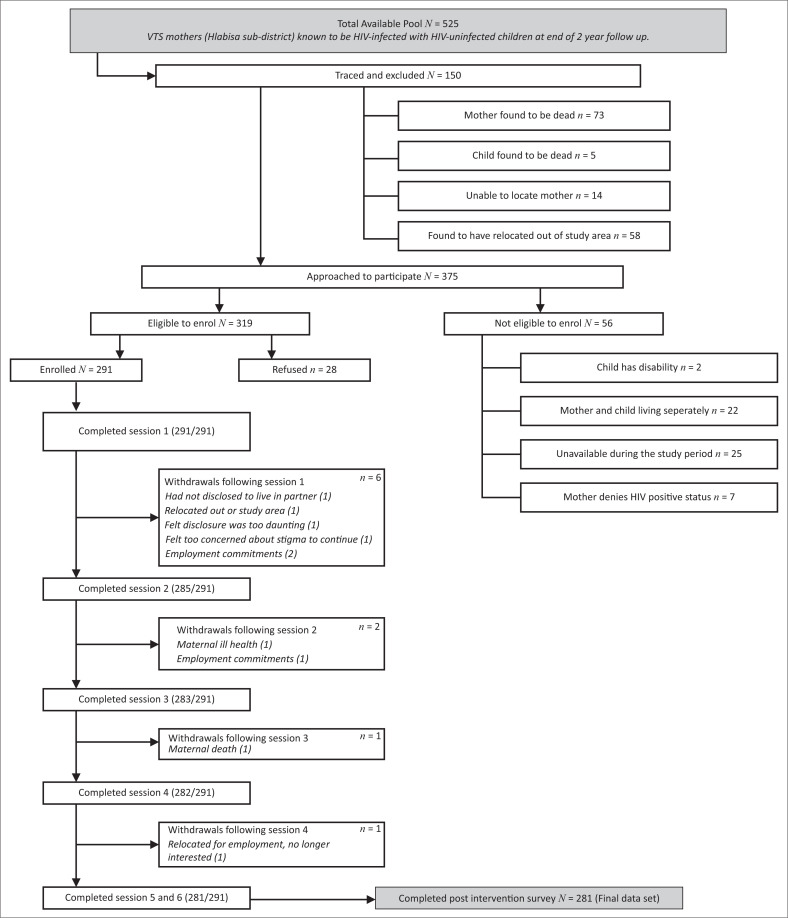
The consort diagram showing women enrolled in the study sourced from and reproduced with permission from the authors.^10^

The sample for the Amagugu evaluation was purposively recruited from an existing cohort in the Vertical Transmission Study (VTS; 2001–2006), a non-randomised intervention study which supported exclusive breastfeeding for the first 6 months post-birth.^33^ Prior participation in the VTS study meant that the mothers’ HIV status during the perinatal period, and hence the child’s HIV exposure status, was known. At the time of VTS, these mothers had given consent to be re-contacted at a later date and for the purpose of this Amagugu study, they were physically traced and invited to participate.^25^ Inclusion criteria were that the mothers were HIV-positive, and their children were HIV-uninfected and between the ages of 6–10 years. In addition, the mother–child pair needed to be in reasonable physical and mental health and reside in the study area. In cases where the mother migrated for work, to be eligible for enrolment, she needed to be staying with her child for a minimum of two nights per week.^10^ The consort diagram is shown in Figure 2. Out of a total available pool of 525 mothers who consented to be contacted at the end of the VTS, 375 women were approached, of whom 291 were enrolled and 281 completed follow-up.

### Data collection

Data in the Amagugu evaluation were collected by questionnaires at 4 time-points: a baseline and post-intervention assessment to collect outcome data. A further two process evaluation semi-structured interviews were completed: the first immediately after disclosure (a post-disclosure interview) and the second conducted 1 week after a health clinic promotion visit (post-clinic visit).^11,25^ This study reports on data from the baseline questionnaires and process evaluation interviews.

During the baseline assessment, data were collected using questionnaires (collected in an interview format) covering information on maternal and child characteristics, including socio-economic, demographic and health information. This included treatment status and CD4 count; partner HIV status and previous HIV disclosure to the index child and family. Process evaluation data included disclosure outcome and type (‘partial’; ‘full’); and post-disclosure questions and reactions of the children. Informational needs of the mothers were derived from the open question of ‘Would you like more storybooks for you and your family? If so, what topics would you like to be covered?’ which was asked in the context of the ‘Family Treasures Story Book’.

At the end-line assessment, questionnaires (collected in an interview format) also collected data on intervention material usefulness including a pre–post evaluation question on whether the ‘Disclosure Safety Hand’ had helped the mothers to talk to their children about the risk of bullying from friends, teacher–child problems, or physical and sexual abuse; whether the participant thought that the ‘HIV Body Map’ could be used to teach about health or sex education. Lastly, information was gathered about whether there were any dolls in the household before the intervention; if the child played with the doll provided by Amagugu; and whether the doll helped the mother to spend more time with her child, listen to her child more and know when her child was worried, happy or excited.

Other data collected in this Amagugu evaluation are detailed and published elsewhere.^25,26,34^

### Analysis

We used a process evaluation design to analyse data which has not previously been analysed.

Data were transformed, coded and analysed in two phases to address each research aim.

In phase 1, we analysed the qualitative data collected through end-line questionnaires on intervention resource use to evaluate which materials in their current form fostered mother–child communication about HIV, health behaviours and sex education. We used descriptive statistics including cross-tabulations and chi-square tests to investigate whether there were significant differences amongst mothers who chose to use the intervention materials, whether those materials were used to discuss health- and sex-related topics with their children, and whether it fostered mother–child communication and maternal attentiveness to the child’s feelings. Where a cross-tabulation contained multiple cells, adjusted standardised residuals were calculated to determine which cells did not differ by chance. The analysis was undertaken using Stata version 13.^35^

In phase 2, we analysed and reported on the process evaluation data collected during the two semi-structured interviews on what other storybook topics the mothers would like to be trained on, to identify additional information that would be useful to mothers. The data were systematically categorised and quantified using content analysis^36^ with the following steps: the third author repeatedly read the data and identified recurrent codes of informational topics requested by the mothers, once an exhaustive codebook was finalised through review by the third and last author to check consistency and saturation of codes, all of the responses were re-analysed and coded. Thereafter the first author independently reviewed the coded data and queries and discrepancies were resolved by consensus between first, third and last authors. Secondly, the codes were then grouped into categories by the first and third author and were reviewed together with the last author. The analysis was conducted using Microsoft Excel. Because some mothers provided more than one response for future storybook topics, a *Z*-test was conducted to determine whether there was a significant difference between the first and all responses. Logistic regression models were computed to test for the effects of maternal characteristics, child characteristics, post-disclosure reactions of children and post-disclosure questions of children on the likelihood of mothers asking for more information on each of the categories derived from the content analysis. Regression models were run controlling for disclosure type (partial; full), with and without controls.

### Ethical consideration

Ethical approval was obtained from the Biomedical Research Ethics Committee (BREC) of the University of KwaZulu-Natal (Ref: BF 144/010).

## Results

### Sample characteristics

Table 1 shows the sample characteristics by post-intervention disclosure level.^26^ All mothers had engaged in some degree of disclosure at that time, with 110 (39%) mothers reporting ‘partial’ and 171 (61%) reporting ‘full’ disclosure to their child.^26^ Almost all had completed at least some primary school education, were in a current relationship and did not have a regular source of income. A large proportion of the mothers were in relatively good health, defined both objectively (having a CD4 count above the eligibility criteria for antiretroviral therapy [ART] at that time of 350 cells/mL) and subjectively (perceiving their current health to be ‘excellent’), although over half of the sample was not yet on ART. The median age of children was 7.0 (interquartile range [IQR] = 7–8) years, and most had a father who was still alive and contributed financially to their care. Disclosure to children prior to the intervention was low.

**TABLE 1:** Sample characteristics.

Characteristics	Partial disclosure (*n* = 110)			Full disclosure (*n* = 171)		
	*n*	%	IQR	*n*	%	IQR
**Maternal characteristics (*n* = 281)**						
Median age, years	33.5	-	29–39	35	–	29–40
**Education**						
No education	7	6.4	-	10	5.9	-
Completed some or all primary	40	36.4	-	68	39.8	-
Completed some or all secondary	59	53.6	-	89.0	52.1	-
Post-school education	4	3.6	-	1	0.6	-
Missing	0	0.0	-	3	1.6	-
**Employment**						
Employed	40	36.4	-	50	29.2	-
Unemployed	69	62.7	-	119	69.6	-
Missing	1	0.9	-	2	1.2	-
**Access to regular remittances**						
No	85	77.3	-	123	71.9	-
Yes	25	22.7	-	48	28.1	-
**CD4 Count (most recent), cells/mL**						
≥ 501	31	28.2	-	40	23.4	-
351–500	20	18.2	-	33	19.3	-
≤ 350	30	27.3	-	47	27.5	-
Missing	29	26.3	-	51	29.8	-
**HIV treatment status**						
Not on ART	60	54.6	-	95	55.6	-
On ART	46	41.8	-	72	42.1	-
Missing	4	3.6	-	4	2.3	-
**Hospitalisation (last 12 months)**						
No	96	87.3	-	154	90.1	-
Yes	14	12.7	-	16	9.4	-
Missing	0	0.0	-	1	0.5	-
**Perceptions of current health**						
My health is excellent	-	-	-	-	-	-
Not true	23	20.9	-	60	35.3	-
True	87	79.1	-	110	64.7	-
**Relationship status**						
No current partner	27	24.5	-	21	12.3	-
Current partner	83	75.5	-	150	87.7	-
**Living with partner**						
No current partner	27	24.6	-	21	12.3	-
Yes	35	31.8	-	57	33.4	-
No	48	43.6	-	92	53.8	-
Missing	0	0.0	-	1	0.5	-
**Partner’s HIV status**						
No current partner	27	24.6	-	21	12.3	-
Unknown status	35	31.8	-	65	38.0	-
HIV positive	34	30.9	-	65	38.0	-
HIV negative	14	12.7	-	20	11.7	-
**Child characteristics (*n* = 281)**						
**Gender**						
Female	55	50.0	-	85	49.7	-
Male	55	50.0	-	86	50.3	-
Median age, years	7	-	6–8	7	-	7–8
**Father still alive**						
No	33	30.0	-	43	25.2	-
Yes	76	69.1	-	127	74.3	-
Missing	1	0.9	-	1	0.5	-
**Father contributes financially**						
Not applicable	34	30.9	-	44	25.7	-
No	30	27.3	-	55	32.2	-
Yes	45	40.9	-	69	40.4	-
Missing	1	0.9	-	3	1.7	-
**Hospitalisation (since birth)**						
No	83	75.5	-	138	80.7	-
Yes	20	18.2	-	23	13.5	-
Missing	7	6.3	-	10	5.8	-
**Prior disclosure to index child**						
No	99	99.0	-	156	91.2	-
Yes – partial disclosure	4	3.6	-	3	1.8	-
Yes – full disclosure	7	6.4	-	12	7.0	-

HIV, human immunodeficiency virus; ART, antiretroviral therapy; IQR, interquartile range.

The descriptive results suggested that the intervention materials in their current form improved parental capacity for health and sex education. Table 2 shows descriptive statistics on whether the ‘Disclosure Safety Hand’ increased mother–child communication during the Amagugu evaluation on the topics of risks of bullying from friends, teacher–child problems and physical or sexual abuse. Overall, the findings indicated that the intervention material did increase communication across all categories. The intervention material facilitated maternal discussion amongst approximately half of the mothers post-intervention who had never engaged in such discussions pre-intervention with their boy children. Child sex was associated with differences for the categories of ‘talked before and talked during’ and ‘did not talk before or during’ on communication about the risks of sexual abuse (*p <* 0.01). The category of ‘talked before and during’ for communication about the risks of physical abuse (*p* < 0.05) also differed by child sex.

**TABLE 2:** Cross tabulation of parental talks on challenging topics with the use of the ‘Disclosurs Hand’.

Discussion outcome	Frequency				Total (*n* = 281)
	Boy (*n* = 141)		Girl (*n* = 140)		
	*n*	%	*n*	%	*n*
**Risks of bullying from friends**					
Did not talk before, talked during	81	57.4	69	49.3	150
Talked before and talked during	54	38.3	67	47.9	121
Did not talk before or during	4	2.8	3	2.1	7
Missing	2	1.4	1	0.7	3
**Teache-child problems**					
Did not talk before, talked during	74	52.5	68	48.6	142
Talked before and talked during	54	38.3	64	45.7	118
Did not talk before or during	11	7.8	7	5,0	15
Missing	2	1.4	1	0.7	3
Physical Abuse*					
Did not talk before, talked during	80	56.7	67	47.9	147
Talked before and talked during	50†	35.5	69†	49.3	119
Did not talk before or during	9	6,4	3	2.1	12
Missing	2	1.4	1	0.7	3
Sexual Abuse**					
Did not talk before, talked during	68	48.2	58	41.4	126
Talked before and talked during	49†	34.8	72†	51.4	121
Did not talk before or during	21†	14,9	8†	5.7	29
Missing	3	2.1	2	1.4	5

† , Significant adjusted standardised residuals.

* , *p* < 0.05,

** , *p* < 0.01.

Most of the mothers reported having used the ‘HIV Body Map’ for sex (*n* = 267; 95.0%), and health education (*n* = 274; 97.5%) with no gender differentials being observed. When reporting on the use of the Amagugu doll, only 94 (33.6%) mothers reported that there had been a doll in the household pre-intervention. After the introduction of the intervention doll, almost all mothers reported that their child had played with the doll (*n* = 270; 96.4%). Most mothers reported that since receiving the doll they had played with their child more (*n* = 264; 94.3%). In relation to whether the doll helped foster mother–child communication, mothers reported that the doll had helped them listen to their child more (*n* = 262; 93.6%), and to know when their child was worried (*n* = 256; 91.4%), or happy or excited (*n* = 257; 91.8%). Encouragingly, the doll was equally popular with both boy and girl children as no gender differences were observed (*p >* 0.05).

In total, 276 (98.6%) mothers responded that they would like more educational storybooks. In response to the question of what topic they would like for future books, 281 mothers gave 363 suggestions, excluding ‘missing’ or ‘not applicable’ responses. These topics were coded and grouped into three main categories and further broken down into three sub-categories: ‘HIV and disease management’ (HIV and TB education and caregiving/disclosure/stigma); ‘General health, safety and health promotion’ (health education/health promotion/sex education); and ‘Family, moral development and aspirations for the future’ (aspirations and family values/morals and social norms/parenting skills).

Table 3 shows categories of story book topics suggested by the mothers. The most popular topics amongst mothers were those relating to ‘Family, moral development and aspirations’. Mothers expressed interest in learning parenting skills with topics such as ‘how to have a good relationship with children’, ‘raising children and how to treat children’ and ‘talk about love as a parent’ being requested for future books. The second most requested category comprised topics related to ‘General health, safety and health promotion’. In this category, requests for books covering health and sex education were prominent with examples, including ‘what to do when you are sick…’, ‘learn about drug abuse’, ‘child abuse and rights’, ‘sexually transmitted diseases’ and ‘encouraging children to talk when they are abused’. The category with the least counts was ‘HIV and disease management’, which covered topics related to the aetiology, prevention and treatment of HIV/TB, maternal and family disclosure, and stigma surrounding illness, with parents’ requests ranging from ‘how HIV is transmitted to babies’, ‘more about disclosing in the family’ and ‘any topics related to not stigmatising someone else’.

**TABLE 3:** Maternal suggestions for additional story book topics for the family.

Categories†,‡	First responses only (*n* = 281)		All responses (*n* = 375)		*Z*(*p*)
	*n*	%	*n*	%	
Category 1: ‘HIV and disease management’	67/281	23.8	79/375	21.1	*Z* = 1.39 *p* = 0.16
HIV/TB education and caregiving	61	91.0	73	92.4	
Disclosure	2	3.0	2	2.5	
Stigma	4	6.0	4	5.1	
Category 2: ‘General health, safety and health promotion’	92/281	32.7	112/375	29.9	*Z* = 1.56 *p* = 0.12
Health education	36	39.1	39	34.8	
Health promotion	22	23.9	23	20.5	
Sex education	34	37.0	50	44.6	
Category 3: ‘Family, moral development and aspirations for the future’	99/281	35.2	161/375	42.9	*Z* = 1.81 *p* = 0.07
Aspirations and family values	36	36.4	55	34.2	
Morals/social norms	55	55.6	89	55.3	
Parenting skills	8	8.1	17	10.6	
No specified topic§	11/281	3.9	11/375	2.9	
Not applicable	2/281	0.8	2/375	0.5	
Missing	10/281	3.6	10/375	2.7	

† , Refer to Appendix 1 for a more comprehensive table which includes example quotations.

‡ , Some mothers gave more than one response, so their first response is recorded as first responses only and all responses refer to the suggested topics without accounting for order.

§ , Stand-alone topic requests that could not be included in any of the other categories.

Maternal and child characteristics, post-disclosure reactions and post-disclosure questions were regressed against the outcome of storybook category. No characteristics were found to be significant predictors of storybook topic selection, and therefore the results were not included in this article.

## Discussion

This study showed that the mothers found the current Amagugu intervention materials to be useful in leading communication with their preadolescant children around HIV and health behaviours which may include sex education as part of reproductive health and HIV prevention.^8^ These results suggest that parents may be able to overcome their expressed discomfort, embarrassment and lack of knowledge on how to engage their children in discussions about sex-related matters with appropriate and user-friendly materials.^8,15^ These results are encouraging because discussing sex-related issues with children is often reported to be a taboo in many settings.^8,15,22^

Building parental capacity for sex education has been identified in the literature as a key method through which increased health education and prevention occurs in the family context.^8,18^ This involves providing parents and caregivers with the necessary practical tools for laying the early foundation in health needed for a positive trajectory over the life course.^18^ The literature suggests that because family, especially parents, plays an important role in the sexual socialisation of children, their role should be capitalised upon when designing programmes to improve the sexual and reproductive health of children and adolescents.^37,38^ Although research on parent–child communication regarding sexuality with younger children is limited, a multi-site study conducted with adolescents in Burkina Faso, Ghana, Malawi and Uganda demonstrated parental influence on adolescents’ sexual and reproductive health.^37^ It is promising that studies in other parts of the world have demonstrated that parents also agree that the basis for sex education should be the home, supplemented by external facilities such as schools.^39^ In a study conducted in a rural area of the United States, 80% of parents believed that the family should provide sex education to children; 94% reported to have talked to their children about sex, and 87% regarded themselves as the primary source of sexual information for their adolescents.^39^

This research is one of the first to demonstrate that South African HIV-positive mothers are willing and interested in being involved in providing such health education (including education on HIV and sexuality) to their preadolescent and adolescent children. Most studies on parent–child communication on sexual issues have been conducted in the United States, Europe and Australia.^8,15^ According to the World Health Organization, studies on parent–child communication on sexual matters in sub-Saharan Africa are limited, but there is a growing literature on this issue.^38^ Our findings align with existing research which suggests that if parents are equipped with adequate support, they can communicate with their children about HIV/AIDS and sexuality matters.^8^ This would, in turn, assist in HIV prevention in young people who may also be exposed to multiple risks as they enter adolescence, including ill-health, depression and substance abuse.^38^

Parent–child communication is a recognised protective factor during the high-risk developmental stage of adolescence, especially concerning HIV infection, and other sexual and reproductive health outcomes.^8,24,37^ The Amagugu evaluation was shown to increase mother–child communication on topics, including the risks of bullying from friends, teacher–child problems, physical abuse and sexual abuse. This finding is important as these childhood events have been linked to adverse outcomes, including behavioural problems,^40^ mental health disorders,^41,42,43^ substance abuse disorders, sexual risk behaviour and increased risk of HIV infection and interpersonal violence,^41^ especially amongst HIV-affected children who are particularly vulnerable to bullying and abuse.^44,45^

It is encouraging to note that in comparison to baseline, mother–child communication increased for all topics. Importantly, we also found that there were gender-specific and significant increases amongst mothers with boys, towards increased education of the potential risk for sexual abuse amongst their boy children. This is an important finding given that a recent national representative cross-sectional study of sexual abuse in South Africa found that 10% of boys and 14% of girls aged between 15 and 17 years reported some sexual victimisation in their lifetime.^46^ This risk of early sexual abuse amongst boys has also been shown in longitudinal research in South Africa to start early.^47^ Thus, intervention such as Amagugu, which encourage communication with boy children about the risk of sexual abuse, has important potential beyond the context of HIV.

Conversely, we found no gender differentials in the use of the ‘HIV Body Map’ for sex education. This is encouraging as the finding suggests that the distribution of educational resources has the potential to make sex education more gender-inclusive, and overcome the accepted norm that the education of boys is often regarded as the responsibility of the father or male caregiver. This is particularly relevant in the context of rural South Africa, not only where patriarchal gender norms pervade but also where the role of education falls to the mother because the father may often be absent from the household.^48^ A plausible explanation for our finding is that the age-appropriate resources boost a mother’s confidence and empowers her to undertake this task.^49^

An important finding was that a simple tool such as a doll could foster parent–child communication and strengthen the parent–child relationship. This is because a doll can build parental capacity by providing an opportunity for interactive play, and insight into a child’s emotions and thoughts, fundamental to capacitating mothers as agents of early prevention.^50^ Based on existing evidence on the usefulness of dolls in counselling with children, it may also provide children with a valuable tool for expressing concerns about emerging risks to which the child is exposed, which they feel afraid to disclose, but may disclose inadvertently through play.^51,52^ For example, illustrating to the parent through projective play concerns about bullying at school or undisclosed sexual abuse.

In contexts of parental HIV in South Africa, there is very little information on what practical support mothers may need to support them in optimising health education with their children.^11,24^ This study provided novel insights into the type of informational needs the mothers themselves identified to be important for their children as they entered into adolescence. This included requests for more information on family, moral development and aspirations for the future, in addition to information on health and sex. This is an original finding as prior evidence has mainly been restricted to its exploration of parent-led educational topics to the domain of sexual and reproductive health.^50,53^ In comparison, our results, which allowed parents to use an open-ended question to define the topics, illustrate the importance of considering a more value-based approach to health education because this emerged as a parental priority and preference. Interventions which are responsive to this parental desire may benefit from increased engagement and motivation amongst parents to undertake what can be a challenging task.

It is important to consider these parental preferences within the context of South Africa and the culture of the study population. The black South African Zulu population have a sense of pride in traditional values which emphasise morality. Broadly, in African cultures, values emphasise collectivist consciousness where the value of the individual (self-achievement) is not as important as the value of one’s relationship with others and how it contributes to a collective or societal achievement, known as ‘Ubuntu’ which represents a moral philosophy of life.^54^ This may explain why mothers considered the moral developmental of children as important requesting material on topics about the importance of family, culture and traditions, as well as education. With regard to the latter, it is also possible that given the South African history of exclusion and discrimination, parents would be particularly motivated to ensure that their children succeed in post-apartheid South Africa.^55^

### Practical implications

The under-utilisation of caregivers in health education and disease prevention in current approaches is a missed opportunity. These findings provide insight into how parental capacities can be strengthened in a cost-effective way which may guide both researchers and policy makers. Targeting mothers in the home context has the potential to be a feasible and cost-effective point of entry for intervention in early prevention of disease. Specific tools may be useful in engaging and encouraging parental education in areas which are traditionally difficult for parents to handle.

### Limitations of the study

Limitations include that sample size was small and context-specific, thereby limiting generalisability. The sample was also restricted to mothers, a practical decision because as mentioned previously, children in rural South Africa are mostly raised by their mothers.^48^ Hence, we are not able to determine whether fathers would find such approaches helpful or appropriate. However, Amagugu is easily adapted to target fathers and other family members^24^ if it were demonstrated to be acceptable to them.

An additional limitation was that because the data in this evaluation study were based on self-report, social desirability bias may have influenced the responses of the mothers regarding health communication and the usefulness of the materials in achieving behavioural change towards increased communication. The Amagugu evaluation is thus limited in its ability to determine the extent to which the intervention materials or activities played a direct role in changing parental behaviours. This is because the data on disclosure and communication outcomes were not measured objectively, and there was no control group. However, the descriptive findings of the analysis do suggest that the intervention materials in their current form helped to improve the mothers’ capacity for health and sex education, contributing to their (the mothers) capacity for making behavioural changes towards increased health communication. It is thus plausible to infer that potentially the content and activities of the package could have led, at least in part, to the increased rates of disclosure and health education that were reported at the end line assessment. This is particularly so because additional research on the Amagugu intervention using a randomised controlled design has since, indeed, demonstrated efficacy in changing parent behaviour towards disclosure and health promotion.^24^

### Recommendations

A long-term follow-up study, when the children in the sample will reach adolescence, would provide further information about whether Amagugu is an effective intervention for HIV prevention in later life. Moreover, studies should be conducted to ascertain whether these findings are replicated in other population settings. National surveys such as the National Attitudes Survey^56^ could help illuminate parental perceptions in this area and determine broader acceptability. A partial economic evaluation should be conducted to make a stronger case for investment in prevention at home.

## Conclusion

This study provided valuable information on what HIV-positive mothers need to support their HIV-uninfected children’s health education as well as the type of interventional resources they found useful in leading communication around health and sex. The capacity of HIV-positive mothers to lead health and sex education in the home should be capitalised on in HIV prevention strategies. The intervention materials were low cost and administered by lay counsellors, which suggests that early prevention does not have to be a costly and complicated endeavour.

## Appendix 1: Supplementary material

**TABLE 1-A1:** Parental suggestions for additional story book topics for the family by category and response.

Categories†	*n*	%	Order of responses per category	Description of categories	Examples of parental statements of education topics for which parent requested additional support
**Category 1: ‘HIV and disease management’**	**79/375**	**21.1**	-	-	-
HIV/TB education and caregiving	73		First response (86.0%); second responses (14.0%)	Group of topics that pertain to knowledge around the HIV and TB virus including transmission, prevention, symptoms and care	About HIV and informing people about HIV To teach children about HIV Signs and symptoms of HIV TB and HIV transmission About HIV how it is transmitted to babies HIV prevention About living positively with HIV How to care for sick people Child behaviour on how to care about people who are HIV positive
Disclosure	2		First response (100.0%)	Topics that pertain to maternal and family disclosure	About disclosure to children More about disclosing in the family
Stigma	4		First response (50.0%); second response (50.0%)	Topics that deal with stigma and associated discrimination	That people with disease should not be discriminated against Any topics related to not stigmatising someone else
**Category 2: ‘General health, safety and health promotion’**	**112/375**	**29.9**	-	-	-
Health education	39		First response (92.0%); second response (8.0%)	Topics that pertain to general health education including improving health literacy about illnesses (apart from HIV and TB) and drug abuse	Topics about health education Teach about health Healthcare Topics about health issues About different illnesses About how a person gets sick Charts to draw all that they have learnt about sick people What to do when you are sick and who should you report to if you hurt yourself Learn about drug abuse
Health promotion	23		First response (100.0%)	Topics that pertain to the promotion of healthy behaviours and the endorsements of lifestyles that facilitate optimal health including nutrition, sport, hygiene and self-care	About advice on how to live healthy Learn about healthy food How to take care of yourself About health and being familiar with clinic How to protect children Topics like cleanliness and other play Playing safely Information about game park animals and about road crossing (safety) about road signs including robots About football
Sex education	50		First response (66.0%); second response (24.0%); third response (8.0%); fourth response (2.0%)	Topics that pertain to general sex education, child abuse and child rights	Child abuse and child rights Encouraging children to talk when they are abused Warning about daily dangers, teaching about not talking with strangers To teach them more about their bodies About stages of growth How a female should behave Sex related education About sexual transmitted diseases
**Category 3: ‘Family, moral development and aspirations for the future’**	**161/375**	**42.9**	-	-	-
Aspirations and family values	55		First response (65.5%); second response (30.9%); third response (3.6%)	Topics that pertain to family values and succeeding in life	Importance of education Know about his future plans About life The importance and the value of a person The importance of family About family, caring and disclosing everything to family Relationships in families How to resolve conflict in family members or in neighbours Caring about people loving people
Morals/social norms	89		First response (62.0%); second response (34.0%); third response (4.0%)	Topics that pertain to culture, religion and moral behaviour	About cultures and traditions To teach about religion Respect a human being How to live and help neighbours About how children should behave
Parenting skills	17		First response (47.1%); second response (52.9%)	Topics that offer caregiver support	Being a good listener Able to communicate with other people If you have a problem, share it Warning about disclosing anything that happens to you How a mother should conduct herself How to have a good relationship with children Raising children and how to treat children Talk about love as a parent

HIV, human immunodeficiency virus.

† , Some mothers gave more than one response, hence total responses to the question was 365 by 271 mothers (excluding missing).
